# Factors influencing dose delivery in rectal cancer radiotherapy using CBCT-guided intensity modulated techniques

**DOI:** 10.1007/s00411-026-01255-2

**Published:** 2026-09-30

**Authors:** Geanina-Mirela Catona, Loredana G. Marcu

**Affiliations:** 1 Faculty of Physics, West University of Timisoara, Timisoara, 300223 Romania; 2 Bihor County Emergency Clinical Hospital, Oradea, 410167 Romania; 3 Faculty of Informatics & Science, University of Oradea, Oradea, 410087 Romania; 4 Allied Health & Human Performance, Adelaide University, Adelaide, SA 5001 Australia

**Keywords:** Image-guided radiotherapy, Organ filling, Dose-volume histogram, Penta-Guide phantom, Mass changes

## Abstract

This study investigates factors that affect dose delivery in rectal cancer radiotherapy and evaluates the resulting differences between planned and delivered treatment doses using intensity-modulated techniques. Twenty patients were included with 140 cone beam computed tomography (CBCT) images. A correlation analysis was conducted to ascertain the impact of detected misalignment on the Penta-Guide phantom on the planning target volumes (PTVs) and volumes of organs at risk (OARs), and whether shifts from CBCT were affected by patient mass changes. To facilitate a comparative analysis and evaluate dose delivery, Intensity Modulated Radiation Therapy (IMRT) and Volumetric Modulated Arc Therapy (VMAT) plans were simulated in the Monaco treatment planning system and further adapted to each CBCT using the Adapt-to-Shape (ATS) function. Moderate to strong correlations between Penta-Guide detected misalignments and the PTVs, bladder and bowel bag were observed, as well as a strong correlation between translational and rotational shifts and patient mass modifications during treatment. Thus, a reduction in target volume coverage was observed in comparison to the initial plans on CT, with VMAT providing a PTV coverage of nearly 95%, while IMRT resulted in an under-irradiated tumour volume with 93.4% coverage for calculated plans on CBCT. Regarding OARs, a slight decrease was observed in parameter values analyzed on CBCT for bladder (up to 5% for V40 with VMAT), while for bowel bag the evaluated parameters were up to 11% higher for IMRT and 10% for VMAT on CBCT. These results suggest that monitoring organ filling, patient mass, and quality assurance (QA)-related misalignments is essential in rectal cancer radiotherapy to minimize dose discrepancies.

**Supplementary Information:**

The online version contains supplementary material available at https://doi.org/10.1007/s00411-026-01255-2.

## Introduction

Rectal malignancies accounted for 343.817 deaths worldwide in 2022, ranking it the tenth most lethal cancer according to the latest international reports (Bray et al. 2024). For intermediate and locally advanced rectal malignancies radiotherapy is employed as the standard neoadjuvant treatment, which is usually followed by surgery (Kim et al. 2017). Modern irradiation techniques, such as Intensity Modulated Radiation Therapy (IMRT) and Volumetric Modulated Arc Therapy (VMAT), allow for a precise dose distribution to the target while simultaneously reducing the dose to adjacent organs at risk (OARs) due to the high dose gradients involved in those techniques (Rosa et al. 2021). However, in case of rectal cancer radiotherapy, both the tumour and the bladder volume fluctuate on a daily basis in accordance with the state of their respective fillings. It is possible that these modifications may result in underdosage of the target volume or an overdose to the surrounding OARs relative to the intended dose distribution (Nugent et al. 2022). The employment of image guidance, enabled by volumetric imaging techniques, has the potential to facilitate a comprehensive evaluation of variations in bladder filling, rectal filling, and bowel bag movement (Wo et al. 2025). This is important because any error in filling status and organ position can compromise the treatment plan and the patient’s clinical outcome. As a result, strict and regular testing is essential to detect and prevent errors before and during treatment (Poppinga et al. 2020).

Cone-beam computed tomography (CBCT) represents the most widely utilized form of advanced image guidance in radiotherapy, facilitating the monitoring of anatomical variations in clinical treatment (Srinivasan et al. 2014). Daily quality assurance (QA) of CBCT imaging systems can be performed using a Penta-Guide phantom, which evaluates registration and correction accuracy by verifying that the system correctly calculates the shifts needed to align a slightly misaligned isocentre to the machine isocentre (Bissonnette et al. 2008). Accordingly, any discrepancies between the anatomy used in the pre-treatment CT plan and the actual anatomy as observed during treatment can be evaluated through CBCT, allowing for the implementation of corrective measures to address these discrepancies.

A review of recent literature on the role of CBCT in rectal cancer radiotherapy demonstrated that monitoring changes in organ geometry as well as positioning errors with CBCT is essential to determine safety margins for tumour volume, with the objective of achieving optimal coverage while protecting OARs (Catona et al. 2024).

The treatment dose delivered can be recalculated based on CBCT imaging, in order to obtain the fractionated dose distribution for patients undergoing radiotherapy (Chang et al. 2023). In the Monaco treatment planning system (TPS), a strategy can be employed to adapt the treatment plan to the actual anatomy (i.e., filling status and location of individual organs) based on CBCT imaging (Kensen et al. 2023). In this context, the employment of the Adapt-to-Shape (ATS) method utilizing segments of the reference plane facilitates the recalculation of doses (Winkel et al. 2019).

The present study investigates factors affecting dose delivery in rectal cancer radiotherapy and evaluates variations between planned and delivered treatment doses using intensity modulated techniques.

## Methods

### Patient characteristics, CT image acquisition and treatment planning

The present study analyzed data from 20 patients with rectal cancer who were treated at the radiotherapy department of the Bihor County Emergency Hospital between 2023 and 2024. An Elekta Synergy Platform linear accelerator, equipped with an Agility multi-lamellar collimator, was utilized in the treatment. Patient simulation was performed using computed tomography (CT), and the acquired planning CT images were used for radiotherapy treatment planning. Prior to each radiotherapy treatment session patient positioning was verified using the Elekta XVI - kV CBCT system. CBCT images were acquired for image-guided verification of the patient’s anatomical and positional setup, allowing any necessary positioning corrections to be performed before treatment delivery. CBCT images were acquired using the pelvis preset with a clockwise gantry rotation from − 180˚ to 180˚ at a speed of 0.5 revolution per minute at detector position M involving the F1 filter, M20 collimator, 660 projection frames, a slice thickness of 3 mm, and a total acquisition time of 120 s.

In order to analyze factors affecting dose delivery and treatment planning-related dosimetric parameters, as well as differences in these parameters between the planned and actually delivered dose, IMRT and VMAT techniques were simulated in the Monaco (version 6.1.4) TPS. In Monaco, the involved Monte Carlo dose calculation algorithm was used for each patient, for 6 MV photon beams with a dose prescription of 50 Gy in 25 fractions.

Patients were positioned according to our department’s internal protocol, in a supine position with their hands crossed over the chest. A special support was used to maintain the appropriate head, knee and foot positions. Patient simulation was undertaken via a Siemens SOMATOM DEFINITION AS 20 CT-Simulator (Siemens Healthineers, Forchheim, Germany).

To ensure optimal treatment outcome, patients undergoing initial CT scans for treatment planning and subsequent radiotherapy were instructed to have a full bladder during all procedures. This was achieved by consuming 500 ml of water 30 min before the simulation and then before each treatment fraction. The tumour volume and OARs were contoured by an attending physician, based on the recommendations of the International Commission on Radiation Units and Measurements (ICRU) reports 62 and 83 (The International Commission on Radiation Units and Measurements 2010).

Patients were weighed on a weekly basis during the treatment period to monitor any alterations in mass that could affect the corrections applied to treatment.

An analysis of the any isocentre misalignment monitored by means of CBCT QA using the Penta-Guide phantom was conducted throughout the treatment period.

The inclusion criteria employed for this study targeted patients treated for rectal cancer during a specified timeframe (i.e., 2023–2024), irrespective of gender or age, with or without chemotherapy. Patients with metallic implants were excluded to avoid assessment interference due to artifacts on CBCT and to facilitate a comparative analysis of treatment techniques and anatomical variations during treatment.

### Investigated factors and statistical analysis

This study evaluated the difference between the predefined misalignment values reported by Bissonnette et al. (Bissonnette et al. 2008) (x = -1.0 cm, y = 1.4 cm, z = 1.2 cm; ± 2 mm tolerance) and the misalignment detected during the course of treatment. The predefined values were used as reference values for assessing the accuracy of CBCT- based registration and correction using a Penta Guide phantom.

Additionally, any couch shifts were quantified against established tolerance parameters using CBCT imaging. Translational corrections through manual repositioning were performed when shifts fell outside the range of ± 0.7 cm, whereas for rotational shifts a range within ± 3 degrees rotation was considered acceptable. In the event that a patient’s rotation exceeded this range, the patient was repositioned accordingly.

In order to establish whether any differences between the predefined misalignment and the detected misalignment influenced PTV and OARs volumes, and whether those differences were influenced by any fluctuations of patient mass, correlations between the misalignment values and patient mass were calculated in relation to the PTV and OARs volumes. Pearson correlation statistical test was used for evaluating the association between two variables. Correlation coefficients with values of ± 0.500–0.700 were considered indicative of a moderate correlation, whereas coefficients with values of ≥ ± 0.700 were considered indicative of a strong correlation. The sign of the correlation coefficient indicated the direction of the association (positive or negative).

For all parameters, comparison of planned CT doses and accumulated CBCT doses was conducted via matched-pairs t-tests. Statistical differences between treatment techniques were also evaluated using matched-pairs t-tests, using a significance level for the p value of < 0.05.

### Treatment plan calculations based on CBCT

Our department’s internal protocol for verifying the positioning of patients utilizing CBCT requires daily assessments during the initial three-day treatment period, followed by weekly evaluations. To determine the radiation dose delivered to OARs and the tumour volume during treatment, CBCT images with correction shifts were exported to the Monaco TPS. A total of 140 CBCT images were subjected to analysis for the purposes of this comparison.

**Fig. 1:**
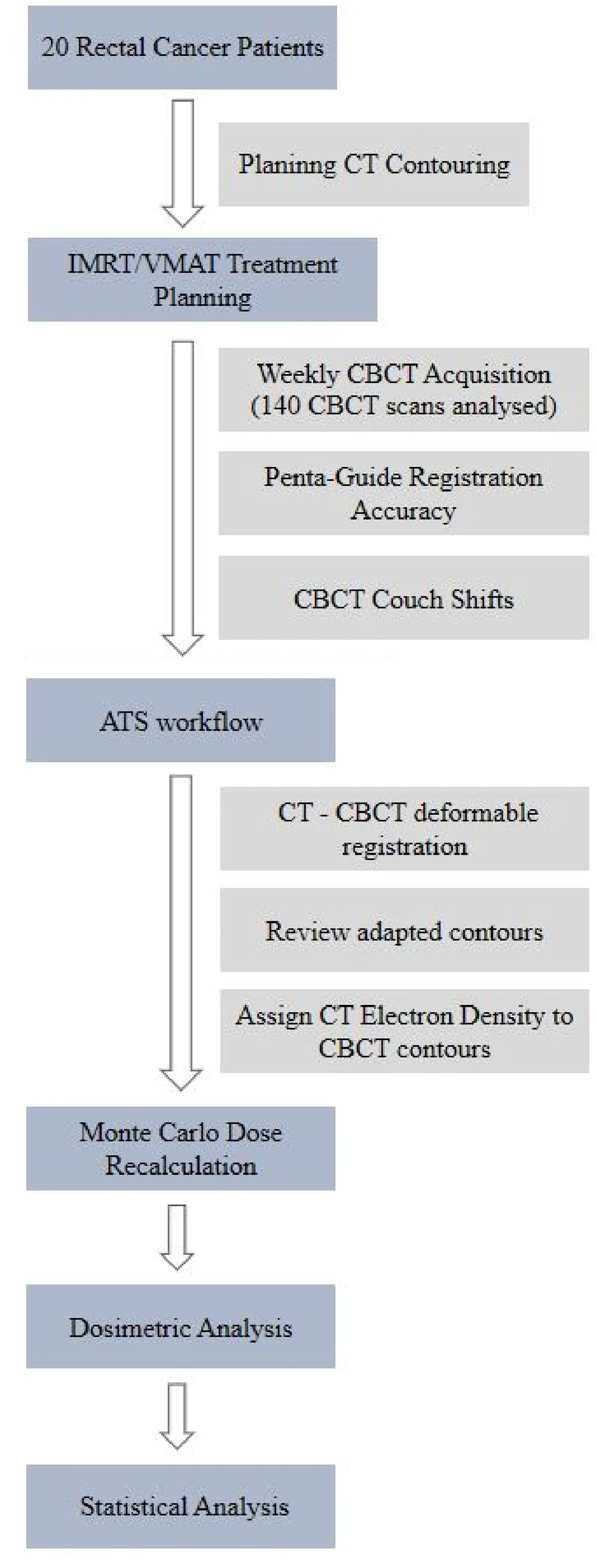
Study workflow. CT – computed tomography; CBCT – cone beam computed tomography; IMRT - Intensity Modulated Radiation Therapy; VMAT - Volumetric Modulated Arc Therapy; ATS - Adapt-to-Shape;

In the Monaco TPS, the original IMRT and VMAT treatment plans generated by the planning CT were transferred to each CBCT using the ATS function. The ATS workflow used deformable image registration to propagate the target and OAR contours from the planning CT to the corresponding CBCT image. The propagated contours were manually reviewed to verify their anatomical correspondence with the CBCT anatomy, and any discrepancies were manually corrected. The mean electron density (ED) value of each structure on the planning CT was assigned to the corresponding contour on the CBCT. This approach provided tissue-density information for CBCT-based dose calculation without relying directly on the Hounsfield unit (HU) values of the CBCT images. The use of CT-based electron density planning information represents one of the approaches described in the literature for CBCT-based dose calculation (Giacometti et al. 2020). During this process, the original beam segments, monitor units (MUs), segment weights and plan isocentre are kept unchanged. The resulting plans on the CBCT images are then recalculated using the Monte Carlo algorithm implemented in Monaco TPS (version 6.1.4), with a dose calculation grid spacing of 0.3 cm and a statistical uncertainty of 1%. In the present study, the same dose calculation settings were used for all CT- and CBCT based dose calculations. The calculated parameters for the PTV and OARs based on the original planning CT were subsequently compared to those based on the corresponding CBCT-calculations. The overall study workflow is shown in Fig. 1.

## Results

### Volume modification of PTV and OARs

**Fig. 2:**
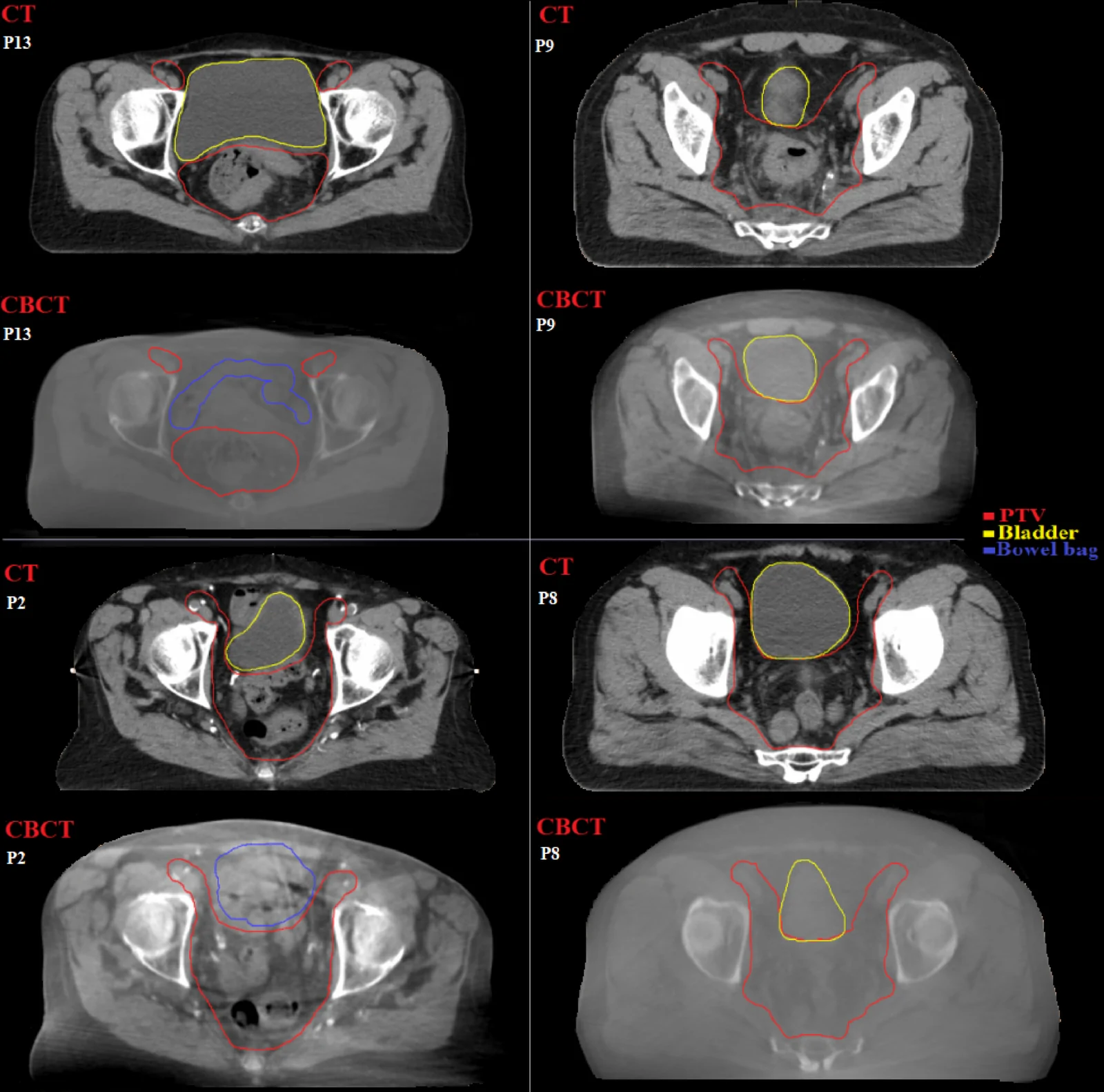
Modifications in planning target volume (PTV), bladder, and bowel bag volumes for patients P2, P8, P9 and P13, illustrated according to the following colour scheme: yellow represents the bladder, red represents the tumour volume, and blue represents the bowel bag

The present findings indicate that there are variations in tumour, bladder, and bowel bag volumes during the course of treatment, as illustrated in Table 1; Fig. 2. The observed variations led to higher radiation doses to OARs and to an underdosage of the tumour volume in comparison with the initially CT-planned IMRT and VMAT doses.

**Table 1:** Mean volumetric changes in the PTV, bladder, and bowel bag (comparison between initial CT-based volumes and actual volumes during radiotherapy as quantified during CBCT. CT – computed tomography; CBCT – cone beam computed tomography; QA – quality assurance; PTV – planned target volume; **Δ =** percentage volume changes

Structure	CT (cm^3^)	Mean CBCT (cm^3^)	Δ (%)
PTV	976.72	957.17	-2.00
Bladder	148.99	136.04	-8.68
Bowel bag	479.67	482.03	0.49

### QA CBCT correlation with PTV / OARs volumes

Moderate to strong correlations were obtained between the misalignment detected with the Penta-Guide phantom during the patients’ treatment, in relation to the volume of the PTV, bladder, and bowel bag.

**Table 2:** Pearson correlation coefficients (r) between the misalignment detected with Penta-Guide phantom and the volume of the planned target volume (PTV), bladder, and bowel bag. Moderate to strong correlations / (positive or negative) are bolded

Patient	*r* - PTV			*r* - bladder			*r* - bowel bag		
	x	y	z	x	y	z	x	y	z
*1*	0.288	-0.161	-0.399	-0.398	**0.589**	-0.338	-0.435	0.043	0.009
*2*	-0.073	**-0.679**	**-0.779**	-0.496	**-0.734**	**-0.925**	**0.621**	**-0.551**	0.286
*3*	0.141	-0.027	**-0.768**	-0.250	**-0.693**	-0.056	0.045	-0.135	-0.143
*4*	0.413	-0.410	**-0.877**	0.359	-0.284	0.292	**0.642**	**-0.592**	-0.149
*5*	-0.373	**-0.560**	**0.837**	0.333	**0.766**	**-0.757**	**-0.631**	**-0.625**	0.327
*6*	**-0.585**	**0.502**	-0.127	-0.341	-0.229	0.068	**0.615**	0.297	-0.450
*7*	-0.472	**-0.881**	0.186	-0.327	**-0.738**	-0.327	0.180	**-0.580**	0.229
*8*	**-0.775**	**-0.778**	0.202	**-0.518**	**-0.660**	-0.333	**0.794**	0.412	0.137
*9*	**0.602**	0.335	-0.408	-0.399	0.199	-0.060	-0.190	-0.071	-0.165
*10*	-0.314	-0.111	-0.050	**-0.531**	**-0.730**	0.467	-0.040	0.383	0.022
*11*	**0.787**	0.356	**-0.827**	-0.409	-0.278	0.416	**0.846**	-0.050	**-0.817**
*12*	0.073	-0.237	0.320	-0.383	0.151	**0.769**	-0.177	0.444	-0.028
*13*	-0.073	**-0.830**	**-0.832**	0.407	-0.265	**-0.922**	0.002	0.232	**0.663**
*14*	**-0.613**	**-0.631**	-0.432	0.218	-0.017	**-0.564**	**0.915**	**0.893**	**-0.570**
*15*	**0.584**	0.262	0.246	-0.130	0.377	-0.270	0.203	-0.491	-0.342
*16*	0.408	-0.030	**0.870**	**0.567**	0.420	-0.368	-0.275	0.345	**-0.673**
*17*	**-0.662**	**-0.570**	-0.466	0.392	0.055	0.116	-0.091	0.109	0.255
*18*	**0.608**	0.219	0.150	**0.758**	**0.934**	0.287	-0.431	-0.423	**-0.975**
*19*	**0.847**	0.410	0.364	**0.842**	**0.893**	0.101	**0.716**	0.281	0.206
*20*	-0.192	**0.699**	-0.211	**-0.630**	0.020	**-0.613**	0.439	-0.190	**0.556**

### Patients’ weight variation and correlations with couch shifts

A decrease in mass was observed in the analyzed patients, with the largest decrease recorded as up to 8 kg (Online Resource 1).

Throughout the five-week treatment period, couch shifts were recorded in all translational and rotational directions. Most shifts remained within the predefined tolerance limits, while some exceeded these limits and required patient repositioning (Online Resource 2).

A moderate to strong correlation was obtained between patient mass changes and couch shifts. This correlation was evident for both translational and rotational shifts, irrespective of the coordinate axis under scrutiny (Table 3).

**Table 3:** Pearson correlation coefficients (r) between patient mass change and couch shifts. Moderate to strong correlations (positive or negative) are bolded

*r*	Translational shifts			Rotational shifts		
*Patient*	*x*	*y*	*z*	*x*	*y*	*z*
*1*	**0.650**	**0.870**	**-0.677**	**-0.543**	**-0.678**	-0.331
*2*	0.214	**0.783**	**-0.590**	-0.272	-0.395	**0.505**
*3*	-0.254	**0.503**	**-0.832**	**0.508**	-0.140	-0.148
*4*	0.031	-0.085	**0.619**	0.214	0.261	**-0.862**
*5*	**0.566**	**-0.529**	**0.747**	**0.943**	-0.258	-0.167
*6*	-0.141	-0.453	0.038	0.184	-0.052	**0.773**
*7*	-0.473	**0.513**	0.391	**0.910**	**0.691**	**0.635**
*8*	0.387	**0.853**	0.465	-0.362	**-0.811**	**0.835**
*9*	-0.373	-0.125	**0.861**	0.225	-0.422	**0.519**
*10*	0.380	**-0.555**	**-0.590**	**0.504**	-1.18	-0.203
*11*	**0.578**	**0.527**	**-0.659**	-0.119	-0.170	0.303
*12*	**-0.772**	0.225	-0.498	0.213	-0.420	**-0.578**
*13*	0.127	**0.647**	**0.896**	-0.232	0.289	-0.201
*14*	-0.399	**0.540**	-0.327	-0.037	**0.572**	-0.413
*15*	-0.408	-0.176	**0.717**	0.100	0.143	**0.570**
*16*	**-0.710**	**-0.571**	**-0.977**	-0.253	0.466	-0.105
*17*	**0.892**	**0.886**	**-0.617**	0.028	**-0.583**	**-0.768**
*18*	-0.499	**-0.535**	0.425	-0.097	**0.744**	-0.304
*19*	**0.597**	**-0.918**	-0.269	-0.141	-0.455	-0.407
*20*	-0.312	**0.603**	**-0.646**	**-0.544**	0.135	0.388

### Planned vs. actually delivered doses to PTV and OARs

**Fig. 3:**
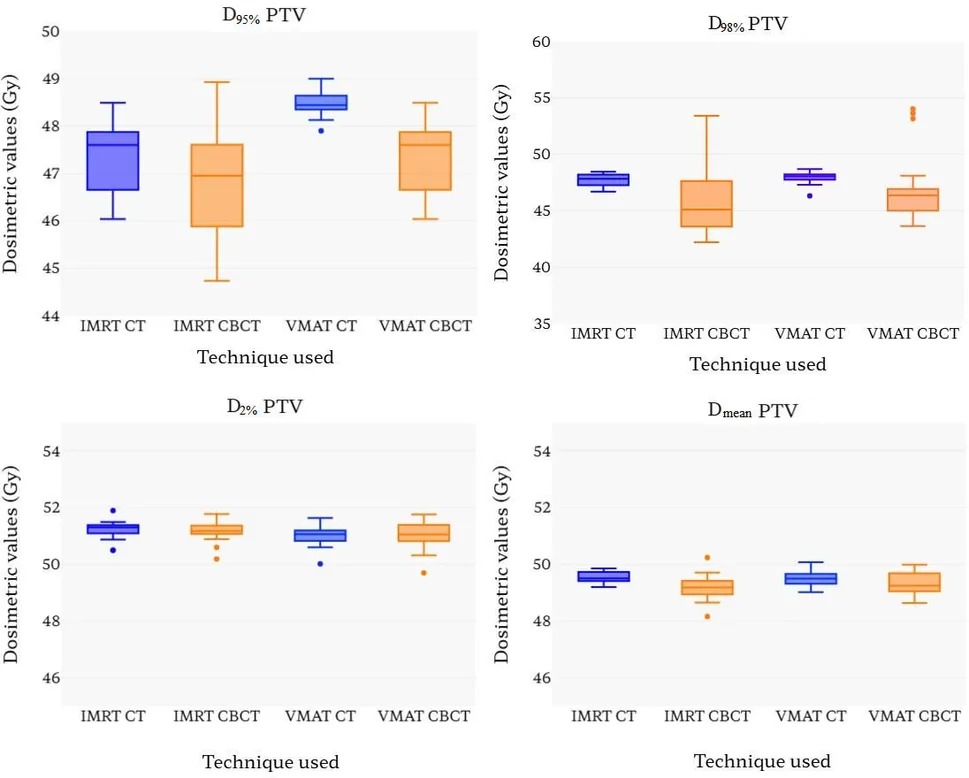
Box plots showing differences between dosimetric values obtained for the planning target volume (PTV) from computed tomography (CT) and cone beam computed tomography (CBCT) for the analyzed parameters.The horizontal line within each box represents the median; the lower and upper limits of the box represent the first (Q1) and third (Q3) quartiles, respectively; the whiskers extend to the most extreme values within 1.5 x the interquartile range (IQR) and the individual dots represent outliers; D_x%_ - minimum absorbed dose that covers x% of the volume of the PTV; IMRT - Intensity Modulated Radiation Therapy; VMAT - Volumetric Modulated Arc Therapy

As compared to the initial treatment planning which was based on CT imaging, a reduction in target volume coverage was observed by means of CBCT imaging as radiotherapy progressed: Specifically, VMAT provided a PTV coverage of nearly 95% (47.41 Gy), while IMRT resulted in an under-irradiated tumour volume with 93.4% coverage (46.70 Gy). The differences between IMRT and VMAT dosimetric parameters (D_95%_, D_98%_, D_2%_, and D_mean_) obtained for the PTV based on CT vs. CBCT images for the whole patient cohort are illustrated in Fig. 3.

**Fig. 4:**
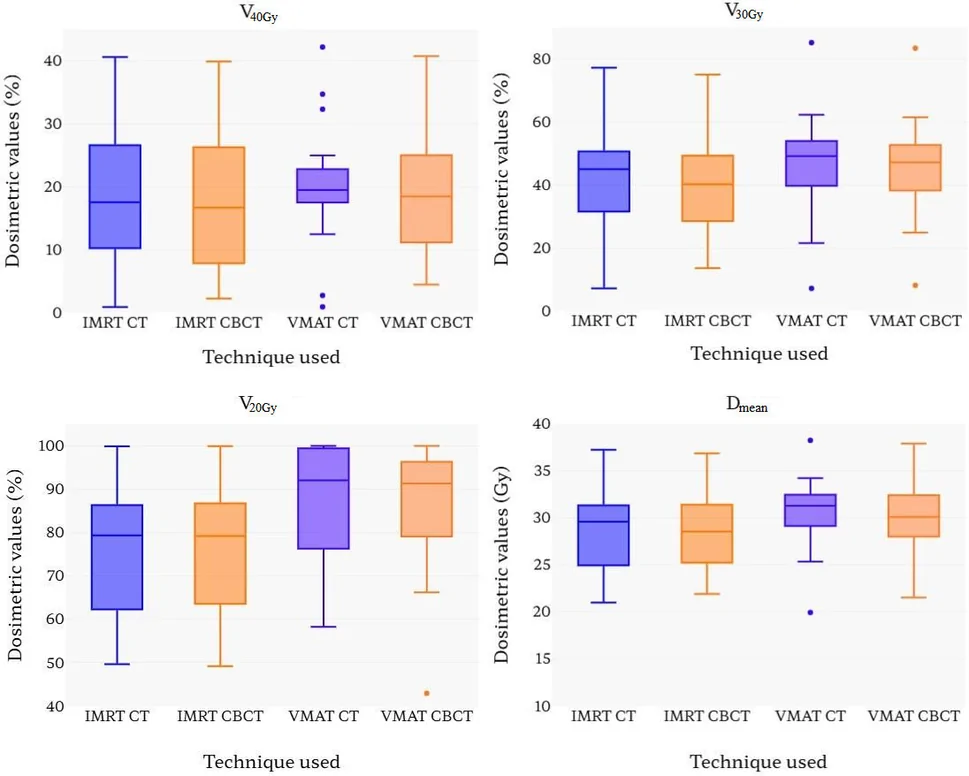
Box plots showing differences between doses obtained for the bladder based on initial computed tomography (CT) image planning and cone beam computed tomography (CBCT) imaging; The horizontal line within each box represents the median; the lower and upper limits of the box represent the first (Q1) and third (Q3) quartiles, respectively; the whiskers extend to the most extreme values within 1.5 x the interquartile range (IQR) and the individual dots represent outliers; V_xGy_ (%) = % volume receiving at least x Gy; IMRT - Intensity Modulated Radiation Therapy; VMAT - Volumetric Modulated Arc Therapy

Regarding OARs, a slight decrease was observed in the parameter values based on CBCT imaging for the bladder, in comparison to the values obtained during treatment planning based on CT imaging. The mean dose was 28.47 ± 4.05 Gy with IMRT and 30.14 ± 3.28 Gy with VMAT based on CBCT imaging, while the mean dose was 28.67 ± 4.09 Gy with IMRT and 30.39 ± 3.82 Gy with VMAT based on initial CT imaging. The discrepancy between the dosimetric values for the OARs obtained during the planning CT session and those obtained through CBCT image analysis is illustrated in Fig. 4.

**Fig. 5:**
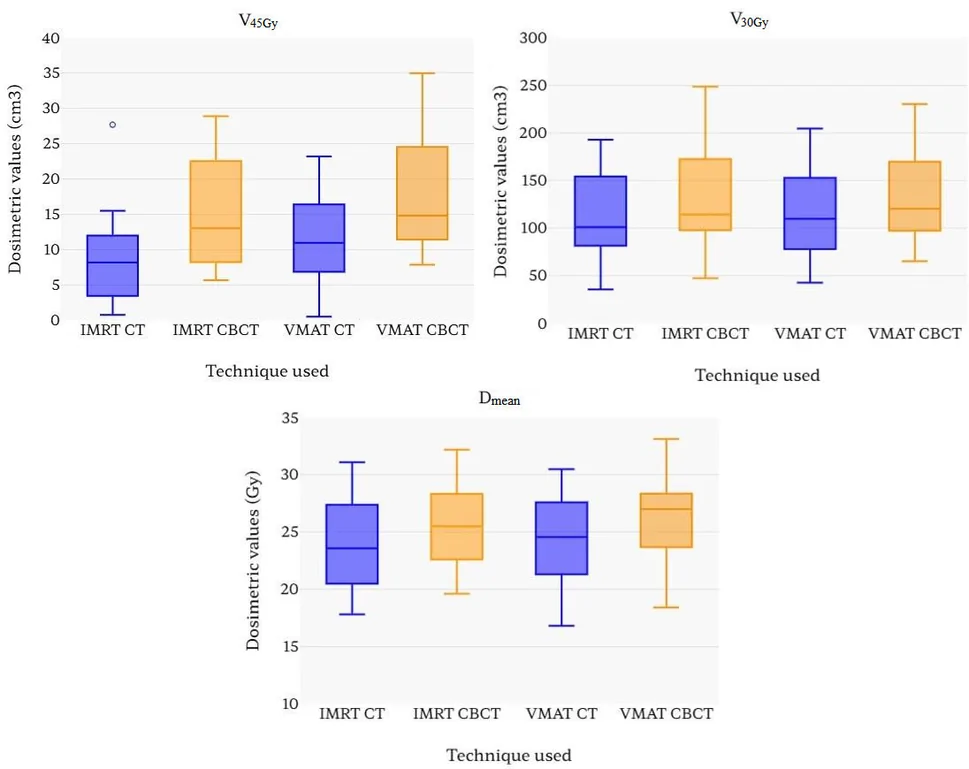
Box plots showing differences between doses obtained for the bowel bag based on initial computed tomography (CT) image planning and cone beam computed tomography (CBCT) imaging; The horizontal line within each box represents the median; the lower and upper limits of the box represent the first (Q1) and third (Q3) quartiles, respectively; the whiskers extend to the most extreme values within 1.5 x the interquartile range (IQR) and the individual dots represent outliers; V_xGy_ (cm^3^) = cm^3^ of volume receiving at least x Gy; IMRT - Intensity Modulated Radiation Therapy; VMAT - Volumetric Modulated Arc Therapy

**Fig. 6:**
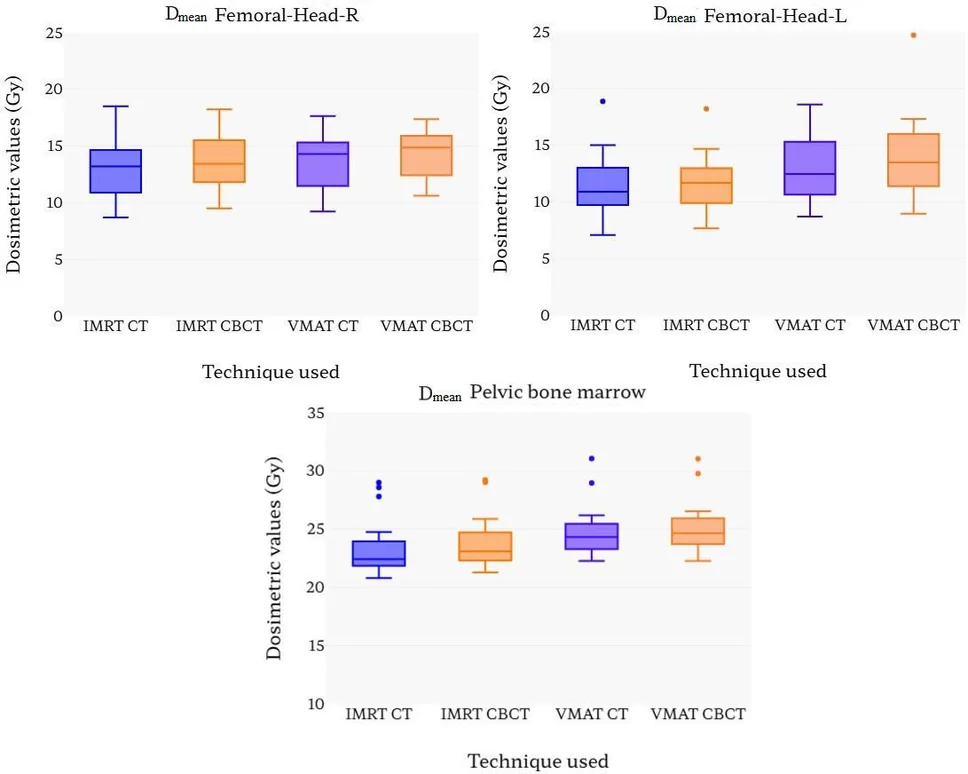
Box plots showing differences between doses obtained for the femoral heads and pelvic bone marrow based on initial computed tomography (CT) image planning and cone beam computed tomography (CBCT) imaging; The horizontal line within each box represents the median; the lower and upper limits of the box represent the first (Q1) and third (Q3) quartiles, respectively; the whiskers extend to the most extreme values within 1.5 x the interquartile range (IQR) and the individual dots represent outliers; IMRT - Intensity Modulated Radiation Therapy; VMAT - Volumetric Modulated Arc Therapy

Dosimetric values obtained for the bowel bag for the analyzed parameters were found to be higher based on CBCT imaging compared to those based on CT image planning (Fig. 5). The mean doses for pelvic bone marrow and femoral heads did not vary significantly between those based on CT image planning and on CBCT imaging (Fig. 6).

The main dosimetric parameters evaluated for PTV coverage and for the OARs obtained during the CT image planning, as well as those calculated based on CBCT imaging are presented in Table 4. A statistically significant difference was observed in PTV coverage between VMAT and IMRT is shown (*p* = 0.001). Additionally, statistically significant differences were also identified with regard to OARs. For instance, for the bladder, the difference between IMRT and VMAT in respect to V_30Gy_ (*p* = 0.009), V_20Gy_ (*p* < 0.001), and D_mean_ (*p* = 0.003) was statistically significant.

**Table 4:** Dosimetric parameters for target volume coverage evaluation and doses to organs at risk. Statistically significant differences are highlighted in bold. IMRT - Intensity Modulated Radiation Therapy; VMAT - Volumetric Modulated Arc Therapy; CT - computed tomography; CBCT - cone beam computed tomography; PTV - Planning target volume; L - left; R - right

Organ		Planning CT			CBCT		
		IMRT	VMAT	*p* value	IMRT	VMAT	*p* value
PTV	*D*_*95%*_ *(Gy)*	48.24 ± 0.39	48.47 ± 0.25	**0.025**	46.70 ± 1.24	47.41 ± 0.73	**0.001**
	*D*_*98%*_ *(Gy)*	47.68 ± 0.59	47.94 ± 0.51	**0.047**	46.19 ± 3.49	46.92 ± 3.13	0.122
	*D*_*2%*_ *(Gy)*	51.22 ± 0.32	51.00 ± 0.35	**0.022**	51.19 ± 0.37	51.00 ± 0.46	**0.041**
	*D*_*mean*_ *(Gy)*	49.54 ± 0.20	49.51 ± 0.26	0.534	49.18 ± 0.43	49.31 ± 0.14	0.147
Bladder	*V*_*40 Gy*_ *(%)*	18.54 ± 10.6	20.17 ± 9.43	0.174	17.98 ± 11.6	18.96 ± 9.16	0.344
	*V*_*30 Gy*_ *(%)*	41.95 ± 16.07	46.62 ± 16.24	**0.005**	40.45 ± 15.7	45.05 ± 14.8	**0.009**
	*V*_*20 Gy*_ *(%)*	75.67 ± 14.7	85.89 ± 14.3	**< 0.001**	75.97 ± 14.3	86.62 ± 14.3	**< 0.001**
	*D*_*mean*_ *(Gy)*	28.67 ± 4.09	30.39 ± 3.82	**0.001**	28.47 ± 4.05	30.14 ± 3.28	**0.003**
Bowel bag	*V*_*45 Gy*_ *(cm* ^*3*^ *)*	12.26 ± 14.6	14.08 ± 13.1	0.255	17.91 ± 17.5	18.81 ± 11.7	0.640
	*V*_*30 Gy*_ *(cm* ^*3*^ *)*	121.6 ± 70.3	131.8 ± 91.3	0.134	135.2 ± 61.8	145.4 ± 8.8	0.153
	*D*_*mean*_ *(Gy)*	23.90 ± 3.89	24.41 ± 3.94	0.092	25.56 ± 3.56	26.25 ± 3.83	**0.045**
Pelvic bone marrow	*D*_*mean*_ *(Gy)*	23.33 ± 2.43	24.75 ± 2.13	**< 0.001**	23.77 ± 2.20	25.09 ± 2.13	**< 0.001**
Femoral-Head-R	*D*_*mean*_ *(Gy)*	13.03 ± 2.59	13.56 ± 2.53	**0.019**	13.63 ± 2.34	14.24 ± 2.20	**0.010**
Femoral-Head-L	*D*_*mean*_ *(Gy)*	11.38 ± 2.70	13.59 ± 4.30	**< 0.001**	11.69 ± 2.33	14.02 ± 3.50	**< 0.001**

## Discussion

In rectal cancer radiotherapy it is widely acknowledged that the position and volume of the rectum, bladder, and bowel bag vary from one treatment fraction to another due to their different filling status (Fig. 2). These volume variations in combination with CBCT-identified misalignments as well as in fluctuations in the patient’s weight result in changes in the actually delivered dose.

The present study showed moderate to strong positive and negative correlation between the misalignment detected with the Penta-Guide phantom during the course of radiotherapy, in relation to the PTV, volume of bladder, and volume of bowel bag (Table 2). Consequently, the observed correlations suggest that these misalignments may affect the delivered dose to the tumour volume and OARs, even if the detected misalignments are within the tolerance (Bissonnette et al. 2008).

Also, a strong correlation was demonstrated between translational and rotational shifts and changes in patient mass during treatment, suggesting that the patient’s position on the treatment couch is influenced by changes in mass (Table 3). In their study, Kim et al. demonstrated a positive correlation between intra-fractional variations and alterations in body mass or body mass indices. Consequently, they proposed that these indices should be considered for accurate IMRT planning in patients with head and neck cancer (Kim et al. 2019). Choi et al. confirmed that changes in body mass during prostate VMAT can result in substantial alterations to target dose distribution (Choi et al. 2017).

Despite the demonstrated efficacy of Penta-Guide in facilitating routine daily quality control of the Image-Guided Radiation Therapy (IGRT) clinical workflow, which encompasses manual or automatic image registrations and automatic treatment table corrections (Poppinga et al. 2020), it is imperative to consider misalignments that arise during the QA process, as these misalignments have the potential to influence the PTV and the volume of the OARs. Consequently, there is a need for further analysis of the changes in mass on dosimetric parameters and treatment outcome.

The translational and rotational shifts analyzed in this study exhibited variability from one week of treatment to another. These shifts were only evident in a single treatment session per week when CBCT was performed (Online Resource 2). It is possible that the remaining sessions, which did not involve CBCT, were similar or even more unfavorable. Seah et al. showed that target shifts even with rotational errors of 5⁰ exert minimal influence on clinical target volume (CTV) target coverage when standard treatment parameters are employed, such as symmetric field size, centric isocentre placement, and single dose level (Seah et al. 2022). Alickikus et al., however, demonstrated that a mesorectal shift of 20 mm laterally and in the anterior/posterior direction and of up to 10 mm in the inferior/upper direction, results in mean dose changes of up to 2% in D_2%_, D_95%_ and D_98%_ (Alickikus et al. 2020).

These variations of translational and rotational shifts in the present study led to an increased number of patients receiving a notably higher dose of radiation to various OARs. Furthermore, in the present study an underdosage of tumour volume was observed based on CBCT imaging in comparison to the values obtained during CT planning for IMRT and VMAT (Fig. 3). The greatest variations in tumour volume coverage were observed for patients P1, P4, P7, P14, and P17 between the doses based on CT image planning those based on CBCT imaging (Online Resource 3a).

These variations can lead to underdosage of tumour volume resulting in loss of tumour control, as well as bladder overdosage resulting in both acute and late toxicity. These findings are in accordance with clinical results previously reported by Yang et al., who analyzed the doses received by the tumour and OARs using CBCT images in cervical cancer radiotherapy. Their results indicated a reduction in PTV coverage compared to the original plan, with the D_98%_ variation of CTV ranging from − 6.59% to 2.18% (Yang et al. 2022). These authors demonstrated that the reduction in tumour volume coverage was attributable to changes in organ volume and shape over the course of treatment. Additionally, Ergen et al. examined inter-fractional organ motion during neoadjuvant radiotherapy for rectal cancer patients using CBCT. Their findings indicated that D_2%_, D_95%_, and D_98%_ doses were influenced by mesorectal motion, with changes up to 2% of the mean dose values (Ergen et al. 2023). These results are comparable to those obtained by the present study, as illustrated in Fig. 3, with D_2%_, D_95%_, and D_98%_ values exhibiting variability across CBCT images.

The dosimetric parameters analyzed in the present study via CBCT for the tumour volume demonstrated variability between IMRT and VMAT treatment techniques. Specifically, D_95%_ for IMRT was 46.7 Gy, versus 47.41 Gy for VMAT plans (*p* ≤ 0.001). A comparison of the two treatment techniques for the delivered dose calculated based on CBCT imaging reveals that overall, VMAT provides better coverage of the tumour volume, nearly meeting the clinically required criterion of 95%.

In a similar study, Zhang et al. obtained 98.89% tumour volume coverage using the full-arc VMAT technique for CBCT-indicated dose in rectal cancer, compared with 94.8% obtained in the present study (Zhang et al. 2023). This discrepancy is likely attributable to changes in organ volume and shape over the course of treatment as well as poor patient compliance with treatment preparation in certain instances. In the present study it was also noticed that the bladder volume was often smaller when it was obtained based on CBCT imaging as compared to the bladder volume obtained during initial CT treatment planning (Table 1.). This means that in course of radiotherapy the treated patient did not consume the amount of water as instructed at the beginning of treatment, or the time between drinking and starting treatment was too short. The difference in the level of bladder filling between the CT planning image and a pre-treatment CBCT image is shown in Fig. 2 (Patients P13 and P2).

Issues regarding bladder filling levels and their dosimetric impact were also investigated by others. For instance, Yang et al. reported bladder volume reductions in cervical cancer patients by up to 93.60% (Yang et al. 2022). In the present study patient P13 demonstrated the most pronounced variation in V_40Gy_ of bladder relative to the pre-treatment value, indicating a notable alteration in bladder volume from one treatment fraction to another (Online Resource 3b). For this patient the V_40Gy_ was 9.68% with IMRT and 12.44% with VMAT during the planning stage. In the first 3 days of treatment V_40Gy_ was 8.2%, 16.85%, 11.45%, respectively, with IMRT and 11.38%, 19.22%, 13.99%, respectively, with VMAT; these values further varied on a weekly basis, up to 74.66% with IMRT and 51.3% with VMAT. While still discrepant, this parameter variation over the first three consecutive days of treatment is small compared to the variation from one week to another, suggesting the important role of daily CBCT imaging.

Given that in in the present study only those changes that may occur during treatment on a weekly basis were assessed, there was no consistency in bladder volume from week to week; thus, the values obtained for dosimetric parameters suggest that the bladder volume already changes on a daily basis. Chauhan et al. in their study on prostate cancer analyzed the bladder filling level using CBCT and ultrasound and showed that there is a progressive decrease in bladder volume from the initial CT planning imaging (194 ml) to subsequent treatments (153 ml) (Chauhan et al. 2023). Thus, it can be concluded that the employment of CBCT on a daily basis is necessary to avoid overdosage of the bladder and, at the same time, the occurrence of late toxicities, such as urinary incontinence, burning and/or other pain during urination.

Similarly, due to the risk of late toxicity associated with pelvic radiotherapy, particularly with regard to late bowel toxicity, it is of great importance to ensure that the bowel bag is as well protected as possible throughout the course of treatment (Jadon et al. 2019). A number of studies have been conducted to determine the optimal dose constraints for the avoidance of these toxicities. For instance, McDonald et al. established that a V_45Gy_ of less than 139 cm³ is associated with a reduced risk of grade 1 bowel bag toxicity in bladder cancer (McDonald et al. 2015). Guerrero-Urbano et al. demonstrated that V_45Gy_ < 71 cm³ can reduce the incidence of grade 2 bowel bag toxicity in patients with prostate cancer (Guerrero-Urbano et al. 2010). The present study revealed that the bowel bag exhibits considerable variation across different treatment fractions, with patient P2 displaying the most pronounced alterations (Online Resource 3c). For this patient V_45_ during planning was 58.85cm^3^ using the IMRT technique and 40.74cm^3^ with VMAT, while V_45_ calculated on the bases of CBCT images varied as follows over the weeks: 134.51cm^3^, 100.48cm^3^, 50.44cm^3^, 61.72cm^3^, 84.04cm^3^, 48.52cm^3^, 119.48cm^3^ for IMRT and 93.29cm^3^, 74.23cm^3^, 36.09cm^3^, 44.12cm^3^, 54.86cm^3^, 32.75cm^3^, 81.26cm^3^ for VMAT. These changes in the dose received by the bowel bag are caused by variations in the volume / position of the PTV relative to the bladder, thus a drop in PTV volume could move the small intestine in the radiation field, receiving a much higher dose than expected.

In terms of doses received by the pelvic bone marrow and femoral heads, there were no significant variations between the CBCT and CT treatment planning scans (Fig. 6).

Regarding the OARs evaluated in the present study, IMRT was associated with lower doses to several OAR parameters compared with VMAT. Statistically significant differences between the two treatment techniques were observed for selected OAR dose parameters on both planning CT and CBCT imaging (Table 4). However, given the relatively small study cohort, these findings should be interpreted cautiously and require confirmation in larger patient populations.

The present study has a number of limitations, including the relatively small sample size of 20 patients. Although this sample allowed for statistical comparison of the dosimetric parameters between IMRT and VMAT, the small cohort limits the generalizability of the findings. Therefore, the observed differences between IMRT and VMAT should be interpreted with caution and validated in larger patient cohorts. Furthermore, dosimetric values for tumour volume and OARs were analyzed only based on CBCT imaging performed during the first three consecutive treatment days, followed by weekly imaging. As CBCT was not performed on a daily basis, the precise radiation dose received by the tumour volume and OARs remains unknown. Additionally, the impact of chemotherapy on tumour volume was not the subject of analysis.

## Conclusions

The present study demonstrates that in the context of radiotherapy for rectal cancer, all volumes involved (of tumour and OARs) undergo changes between treatment fractions, due to variations in their filling state and to factors that affect dose delivery. This can lead to unwanted effects such as underdosage of tumour volume and overdosage of OARs. The greatest differences between the administered treatment dose as calculated based on CBCT imaging and initial planned dose based on CT imaging were observed in the tumour volume and bladder.

It is concluded that close monitoring of body mass during radiotherapy is imperative, as fluctuations in body mass can significantly impact translational and rotational shifts. Therefore, attention should be given to misalignments occurring throughout the QA procedure, as these may impact the PTV and volume of OARs.

## Electronic Supplementary Material

Below is the link to the electronic supplementary material.

Supplementary file 1

Supplementary file 2

Supplementary file 3

Supplementary file 4

Supplementary file 5

Supplementary file 6

## Data Availability

The data that support the findings of this study are available from the corresponding author upon reasonable request.
