# Supplementary material for: Factors influencing dose delivery in rectal cancer radiotherapy using CBCT-guided intensity modulated techniques
Source: Radiat Environ Biophys. 2026 Sep 30;65(4):1099–109. doi: 10.1007/s00411-026-01255-2 (PMC13687293; doi:10.1007/s00411-026-01255-2)
Supplement: Supplementary file 2 [file 411_2026_1255_MOESM2_ESM.docx]

**Online Resource 2**

Geanina-Mirela Catona, Loredana G. Marcu

**Factors influencing dose delivery in rectal cancer radiotherapy using CBCT-guided intensity modulated techniques**

Geanina-Mirela Catona (🖂)

West University of Timisoara, Faculty of Physics, 300223, Timisoara, Romania

Phone: +40 741778129

Email: geanina.catona@e-uvt.ro

ORCID ID: 0009-0007-2859-8728

Geanina-Mirela Catona

Bihor County Emergency Clinical Hospital, Oradea 410167, Romania

Loredana G. Marcu

Faculty of Informatics & Science, University of Oradea, Oradea 410087, Romania

Loredana G. Marcu

Allied Health & Human Performance, Adelaide University, Adelaide SA 5001, Australia

**Fig 2 Online Resource 2** Couch shifts within the recommended limits (translational -0.7 and +0.7 cm; rotational -3 and +3 degrees) and above for the patient group.
