# Supplementary material for: Factors influencing dose delivery in rectal cancer radiotherapy using CBCT-guided intensity modulated techniques
Source: Radiat Environ Biophys. 2026 Sep 30;65(4):1099–109. doi: 10.1007/s00411-026-01255-2 (PMC13687293; doi:10.1007/s00411-026-01255-2)
Supplement: Supplementary file 3 [file 411_2026_1255_MOESM3_ESM.docx]

**Online Resource 3**

Geanina-Mirela Catona, Loredana G. Marcu

**Factors influencing dose delivery in rectal cancer radiotherapy using CBCT-guided intensity modulated techniques**

Geanina-Mirela Catona (🖂)

West University of Timisoara, Faculty of Physics, 300223, Timisoara, Romania

Phone: +40 741778129

Email: geanina.catona@e-uvt.ro

ORCID ID: 0009-0007-2859-8728

Geanina-Mirela Catona

Bihor County Emergency Clinical Hospital, Oradea 410167, Romania

Loredana G. Marcu

Faculty of Informatics & Science, University of Oradea, Oradea 410087, Romania

Loredana G. Marcu

Allied Health & Human Performance, Adelaide University, Adelaide SA 5001, Australia

**Fig. 1, Online Resource 3** Variation of PTV D95% (a), bladder V40Gy (b) and bowel V45Gy (c) for all patient plans simulated with IMRT and VMAT. IMRT - Intensity Modulated Radiation Therapy; VMAT - Volumetric Modulated Arc Therapy; Dx% - minimum absorbed dose that covers x% of the volume of the PTV; V_x Gy_ (%) = % volume receiving at least x Gy; V_x Gy_ (cm^3^) = cm^3^ of volume receiving at least x Gy, PTV – planning target volume.
